# Effect of Butyric Salt Supplementations, as Metabiotics, on Rumen Functions in Pre-Weaned Dairy Calves

**DOI:** 10.3390/life15121820

**Published:** 2025-11-27

**Authors:** Konstantinos V. Arsenopoulos, George Nikolaou, Elias Papadopoulos

**Affiliations:** 1 Department of Veterinary Medicine, School of Veterinary Medicine, University of Nicosia, Engomi, 2414 Nicosia, Cyprus; nikolaou.g@unic.ac.cy; 2 Laboratory of Parasitology and Parasitic Diseases, School of Veterinary Medicine, Faculty of Health Sciences, Aristotle University of Thessaloniki, 54124 Thessaloniki, Greece; eliaspap@vet.auth.gr

**Keywords:** metabiotics, butyric salts, rumen functions, feed intake, growth performance, hematological parameters, histomorphological findings, *Eimeria* spp., *Giardia* spp.

## Abstract

Rumen development is critical for optimal growth, feed efficiency and health in pre-weaned dairy calves. Butyric acid, a short-chain fatty acid produced in the rumen, has been shown to stimulate epithelial development and modulate immune responses. This study evaluated the effects of butyric salt supplementations, as metabiotics, on rumen morphometry and investigated the potential associations with feed intake, growth performance and health indicators in pre-weaned dairy calves. Thirty calves were randomized into three groups: controls (G1), 25 g (G2) or 50 g (G3) sodium butyrate from birth until weaning. The gross and histological rumen morphometry, dry matter intake, growth performance, complete blood count, total protein determination and fecal (oo)cyst counts for *Giardia* spp. and *Eimeria* spp. were analyzed using one-way ANOVA. When significant effects were detected, treatment means were compared using Tukey’s HSD at *p* ≤ 0.05. Sodium butyrate supplementation produced dose-dependent responses. Compared with controls (*p* ≤ 0.05), G3 calves had a greater mean papillae height (3.20 vs. 1.36 mm) and rumen wall thickness (7.08 vs. 3.62 mm), higher final body weight (93.9 vs. 80.1 kg), increased daily weight gain (798 vs. 550 g/day), increased total dry matter intake (69.4 vs. 55.1 kg) and improved feed conversion ratio (1.45 vs. 1.67). In addition, G3 calves showed reduced *Giardia* and *Eimeria* (oo)cyst excretions and favorable hematological shifts at weaning (i.e., higher hematocrit, hemoglobin and mean corpuscular hemoglobin concentration and altered leukocyte differentials) consistent with improved health. In conclusion, sodium butyrate supplementation in pre-weaned dairy calves resulted in dose-dependent improvements in rumen morphometry, voluntary dry matter intake and growth performance. These physiological benefits were accompanied by decreased *Giardia* spp. and *Eimeria* spp. (oo)cyst shedding and by positive shifts in hematological health indicators, suggesting a potential enhancement of the animals’ immune response.

## 1. Introduction

Calf performance in dairy production systems is mainly determined by the effectiveness of nutritional and feeding management practices [1]. Calves represent a strategic investment, contributing to herd replacement, genetic improvement and long-term economic efficiency. The pre-weaning phase is particularly critical, as it lays the foundation for optimal growth and health. Achieving these goals requires not only an adequate nutrient supply but also a clear understanding of digestive physiology and the implementation of feeding strategies that promote gastrointestinal development and resilience [1].

Neonatal calves differ substantially from mature cattle in both the morphology and function of the gastrointestinal tract. While the structural components of the ruminant stomach are present at birth, the rumen, reticulum and omasum remain functionally immature, as reflected in histomorphological findings. In contrast, the esophageal groove is fully operative, and the abomasum demonstrates advanced enzymatic activity, enabling the calf to function primarily as a monogastric animal during the early postnatal period. Consequently, whole milk or milk replacer serves as the principal nutrient source until the introduction of solid feed stimulates rumen growth and functional maturation, facilitating the utilization of fibrous feed resources [2,3]. The early introduction of solid feed, beginning in the first week of life, is therefore essential to support the transition from pre-ruminant to a fully developed ruminant [4].

A central objective of early-life nutritional management is the promotion of rumen papillae development, as papillary morphology is closely associated with digestive efficiency through its impact on the absorptive surface area [5]. The transition from liquid to solid feed, as well as the composition of the diet, exerts a decisive influence on gastrointestinal development, particularly rumen epithelial growth, by inducing a series of coordinated anatomical and physiological adaptations [6,7]. Effective management during the pre-weaning period is therefore of paramount importance, as an insufficient rumen development or inadequate solid feed intake at weaning may constrain subsequent growth and performance [2]. Furthermore, cattle with a superior feed efficiency have been reported to exhibit a more developed rumen epithelium compared to less efficient counterparts [8].

Diet composition, whether forage- or concentrate-based or formulated as a balanced ration, exerts a decisive influence on rumen fermentation processes. The microbial degradation of carbohydrates generates volatile fatty acids (VFAs), particularly butyric and propionic acids, which are key stimulators of epithelial proliferation and differentiation. Epithelial development occurs alongside the growth of rumen musculature, enhanced peristaltic function and increased rumen volume [9].

Towards this direction, dietary supplementation with metabiotics has been reported to promote rumen health and improve growth performance in calves [10]. Metabiotics are defined as bioactive compounds of microbial origin, including metabolites, cell components and signaling molecules, which exert beneficial effects on the host by modulating metabolic, immunological or physiological functions, without requiring the administration of live microorganisms [11]. Among these, butyric salts, such as sodium and calcium butyrate, are the salt derivatives of butyric acid, a short-chain fatty acid produced by microbial fermentation. In adult ruminants, butyrate originates mainly from the fermentation of dietary fiber (i.e., fiber-degrading microbes convert plant polysaccharides into butyric acid) in the rumen. However, in pre-ruminant and weaning calves, the rumen microbiota and fibrolytic activity are still developing, and butyrate production arises predominantly from the fermentation of starch and other readily fermentable carbohydrates present in concentrates. Supplementation with butyric salts provides an exogenous source of butyrate that supports epithelial development and gut health [12]. More precisely, this supplementation accelerates rumen papillae development, enhances absorptive capacity and supports immune modulation, thereby facilitating the transition from liquid to solid diets and improving the overall growth performance [13,14].

Despite the recognized benefits of butyric salts in promoting rumen epithelial growth, nutrient absorption and growth performance, there is limited information regarding their effects on rumen morphology, feed intake, growth and associated health parameters under on-field conditions, particularly in pre-weaned dairy calves. Therefore, the present study was undertaken to evaluate the effects of butyric salt supplementations, as metabiotics, on rumen physiology, feed intake, growth performance and key health indicators, including histomorphological, hematological and parasitological parameters in pre-weaned *Holstein* calves.

## 2. Materials and Methods

### 2.1. Herd and Animals

The present study was conducted in an intensively managed *Holstein* dairy herd, consisting of 500 lactating cows. Routine vaccination protocols included a single dose against rotavirus, coronavirus and *Escherichia coli* K99 (Rotavec Corona^®^, MSD Animal Health, Trenton, NJ, USA), as well as against clostridial diseases (Cubolac^®^, Virbac Hellas, Athens, Greece) administered one month before parturition for both heifers and cows. Additionally, animals were vaccinated biannually against bovine viral diarrhea (BVD) and infectious bovine rhinotracheitis (IBR) using Bovilis^®^ (MSD Animal Health, Rahway, NJ, USA). No clinical cases of mastitis or uterine infections were recorded in the cows during the study period.

Calves were removed from their dams immediately after birth and housed individually. Within the first 2 h post-partum, they received 4–5 L of high-quality colostrum, as assessed by a colostrum meter (Agridirect, Dublin, Ireland). During the first week, calves were fed bulk tank milk, gradually replaced by a commercial milk replacer (Sprayfo Delta^®^, Trouw Nutrition, Berlin, Germany) until weaning at approximately 60 days of age, according to the feeding schedule presented in Table 1. Concentrates were introduced during the first week of life, roughages (chopped alfalfa hay) were provided after the first month (31 days of age) and fresh water was available ad libitum.

### 2.2. Experimental Design

The study was conducted from May to August 2025 on a population of 30 pre-weaned *Holstein* dairy calves. The farm was selected based on convenience, i.e., the willingness of the farmer to collaborate and receive regular visits for examination, sampling and slaughter of the animals included in the present study. All animals were female, clinically healthy and housed under uniform management conditions. These calves were randomly allocated into three experimental groups (*n* = 10 per group) according to the following principles: G1 group—control group: received a standard diet consisting of colostrum, whole milk or milk replacer, alfalfa hay as the sole roughage source and a commercially available concentrate (crude protein 18%, crude fiber 10%, fat 2.5%, moisture 10% and ash 8%) without butyric salt supplementation; G2 group—25 g butyric salts: received the standard diet supplemented with 25 g of butyric salts per animal per day; G3 group—50 g butyric salts: received the standard diet supplemented with 50 g of butyric salts per animal per day. Rumen-targeted butyric salts, consisting of sodium butyrate in a commercially available feed-grade preparation, were administered once daily in the morning feed from birth (Day 0) until weaning (Day 60). The butyric salts were thoroughly mixed with the concentrate to ensure homogeneity and adequate consumption.

### 2.3. Quantification of Feed Consumption

Daily consumption of concentrates and roughages was recorded for each calf and calculated by subtracting refusals from the amount offered after 24 h. Average daily feed intake was expressed in terms of dry matter (DM, kg) for each sampling occasion.

Milk replacer was provided according to the farm’s routine artificial suckling program, with predefined volumes for each calf (Table 1). Each liter of the milk replacer was prepared by mixing 130 g milk powder with 870 mL of warm water.

### 2.4. Determination of the Calves’ Body Weight

All calves were weighed on the day of birth (Day 0) and every 15 days (Days 15, 30, 45 and 60) until weaning. Weighing was performed using a verified scale (Gallagher H.D. Cattle weigh scale feet kit, Gallagher Group, Wellington, New Zealand).

### 2.5. Blood Sampling and Determination of the Complete Blood Count

Blood samples were collected from each calf via jugular venipuncture into 4 mL EDTA-coated vacuum glass tubes (BD Vacutainer^®^, Becton, Dickinson and Company, Plymouth, UK) on Days 30 and 60.

In every blood sample taken, the erythrocyte parameters (i.e., red blood cells—RBC, hematocrit—PCV, hemoglobin—HGB, mean corpuscular volume—MCV, mean corpuscular hemoglobin—MCH, mean corpuscular hemoglobin concentration—MCHC and red cell distribution—RDW) and the megakaryocyte parameters (i.e., platelets—PLT and mean platelet volume—MPV) were determined with the automatic hematological analyzer Mythic^®^ 18Vet (Orphee S.A., Plan-les-Ouates, Geneva, Switzerland). The leukocyte parameters, including white blood cell counts (WBC) and their differential leukocyte counts (i.e., neutrophils—NEUT, lymphocytes—LYM, monocytes—MONO, eosinophils—EOS, basophils—BASO and large unstained cells—LUC), were analyzed using blood smears prepared immediately after sampling. The smears were stained with Giemsa and examined manually under an optical microscope.

### 2.6. Determination of Total Proteins

Plasma total protein (TP) concentrations were measured using an Atago^®^ T2-NE clinical refractometer (Atago Ltd., Tokyo, Japan), following the manufacturer’s instructions.

### 2.7. Fecal Sampling and Parasitological Procedures

Fecal samples were collected from each calf on Day 0 (birth) and every 15 days thereafter (Days 15, 30, 45 and 60) until weaning. Sampling was performed directly from the rectum using single-use plastic gloves and lubricant to ensure hygiene and minimize discomfort. The samples were stored and transported in isothermal containers (maintained at +2–+4 °C) to the Laboratory of Parasitology and Parasitic Diseases, School of Veterinary Medicine, Faculty of Health Sciences, Aristotle University of Thessaloniki. All procedures were carried out by a trained veterinarian in accordance with animal welfare regulations to minimize stress and ensure ethical handling.

Parasitological examination was performed using the flotation technique to detect the protozoan parasites *Eimeria* spp. and *Giardia* spp., which are common enteric protozoa in calves, strongly affecting gut health and weaning performance. *Eimeria*-positive samples were quantified using the modified McMaster method [15], with a sensitivity of 50 oocysts per gram of feces (OPG). *Giardia*-positive samples were evaluated semi-quantitatively based on cyst counts per optical field at 20× magnification, according to the Utaaker [16] recommended scoring system: [score: +1] 1–9 cysts, [score: +2] 10–50 cysts, [score: +3] 51–100 cysts and [score: +4] >100 cysts.

### 2.8. Rumen Tissue Sampling and Histomorphological Examination

At weaning, calves were processed at a local abattoir under the supervision of trained personnel. During postmortem examination, tissue samples approximately 1 cm^2^ in size were collected from four anatomically distinct regions of the rumen: the dorsal rumen sac, ventral rumen sac, dorsal caudal blind sac and ventral caudal blind sac. Gross morphometric measurements, including rumen papillae dimensions and the thickness of the rumen tissue layer, were initially measured using a millimeter ruler to provide baseline anatomical data.

For histomorphological evaluation, rumen tissue samples were processed using standard paraffin-embedding techniques. Tissues were fixed in 10% buffered formaldehyde for 48 h, dehydrated through a graded ethanol series (80%, 96% and 100%) and cleared with three stages of xylene in an automated tissue processor. Following paraffin infiltration, samples were embedded in paraffin and sectioned using a microtome to obtain slices 4 µm thick. Sections were mounted on slides and stained with hematoxylin and eosin. For each sample, three sections exhibiting optimal structural integrity were selected for detailed observation under an optical microscope at 100× magnification. These sections were analyzed to assess papillary morphology, epithelial development and overall rumen tissue organization, providing both quantitative and qualitative measures of rumen maturation.

### 2.9. Statistical Analysis

Data on growth performance, feed intake, rumen morphometry, histological measurements, hematological indices and parasitological parameters were analyzed using one-way analysis of variance (ANOVA), with each calf considered as the experimental unit [17]. Prior to analysis, data were examined for normality and homogeneity of variance using the Shapiro–Wilk and Levene’s tests, respectively. When significant differences were detected, post hoc comparisons among treatment groups were conducted using Tukey’s honestly significant difference (HSD) test. Differences were considered statistically significant at *p* ≤ 0.05. All analyses were conducted using SPSS software (version 2008, SPSS Inc., Chicago, IL, USA).

## 3. Results

### 3.1. Zootechnical Findings

**Figure 1:**
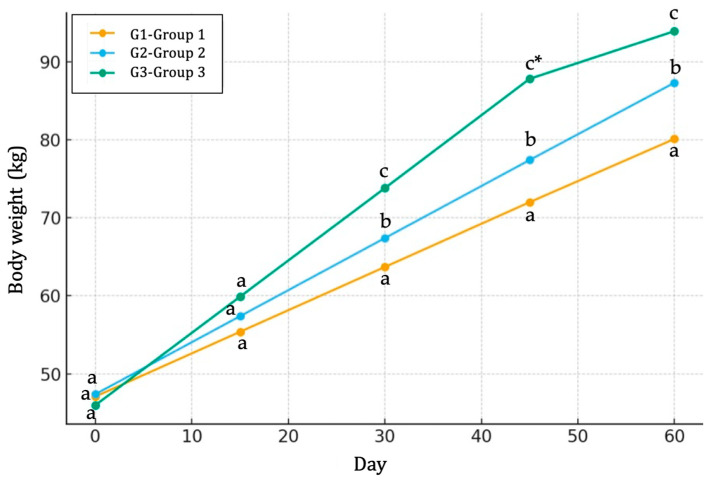
Line charts indicating the evolution and differences in mean body weight (kg) among three groups (G1, G2 and G3) of calves across the experimental study. G1: group of calves (*n* = 10) fed with the standard diet; G2: group of calves (*n* = 10) fed with the standard diet plus 25 g of butyric salts; G3: group of calves (*n* = 10) fed with the standard diet plus 50 g of butyric salts. a, b and c: different letters indicate statistical differences at *p* ≤ 0.05 among groups. * indicates statistical differences at *p* ≤ 0.001 among groups.

Dietary supplementation with butyric salts had significant effects on growth performance, nutrient intake and feed efficiency (Table 2). The initial body weights did not differ among the three groups (Figure 1). However, by 60 days of age, calves receiving butyric salts (G2 and G3 groups) had significantly higher (*p* ≤ 0.05) final body weights compared to the control calves (G1 group), with the greatest (*p* ≤ 0.05) increase observed in the G3 group (Figure 1). Similarly, the daily body weight gain was lowest (*p* ≤ 0.05) in the control group (G1, 550 g/day) and highest (798 g/day, *p* ≤ 0.001) in the G3 group (Table 2).

**Figure 2:**
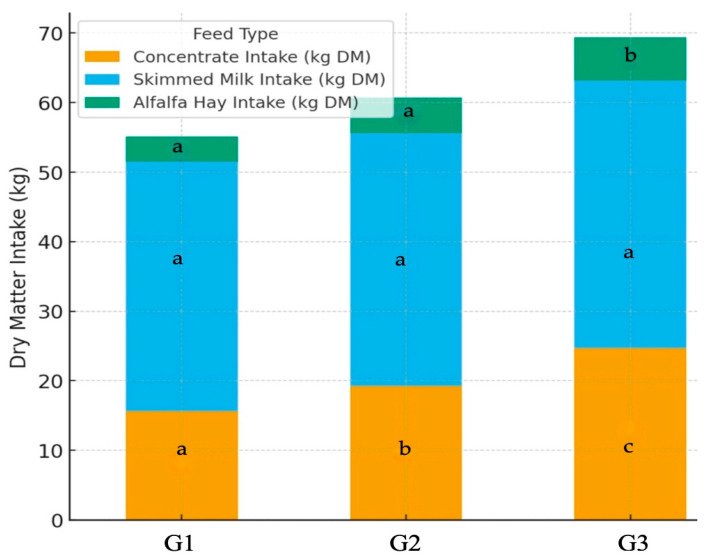
Bar charts indicating the differences in the dry matter (DM) intake (kg) per feed type among three groups (G1, G2 and G3) of calves across the experimental study. G1: group of calves (*n* = 10) fed with the standard diet; G2: group of calves (*n* = 10) fed with the standard diet plus 25 g of butyric salts; G3: group of calves (*n* = 10) fed with the standard diet plus 50 g of butyric salts. a, b and c: different letters indicate statistical differences at *p* ≤ 0.05 among groups.

The dry matter (DM) intake from both concentrates and roughages (i.e., alfalfa hay) increased (*p* ≤ 0.05) progressively with higher levels of butyric salt supplementation (Figure 2). The total DM intake was higher (*p* ≤ 0.05) in the G3 group compared with G1 and G2, respectively. Finally, the feed conversion ratio (FCR) improved significantly with butyric salt supplementation (Table 2), decreasing from 1.67 in the control group to 1.45 in the G3 group (*p* ≤ 0.001).

### 3.2. Hematological Findings

The statistical analysis of hematological data on Day 30, coinciding with the introduction of roughages into the standard diet of the participated animals, showed that calves receiving 50 g of butyric salts (G3 group) recorded the highest (*p* ≤ 0.05) values of PCV, NEUT, MONO, BASO and TP (Table 3) compared with calves in the G2 (i.e., calves receiving 25 g of butyric salts) and G1 (i.e., control calves) groups. On the contrary, EOS values were highest (*p* ≤ 0.05) in the control group (G1 group) compared to G2 and G3 groups (Table 3).

On the day of weaning (Day 60), calves receiving 50 g of butyric salts (G3 group) recorded the highest (*p* ≤ 0.05) values of PCV, HGB, MCHC and MONO compared with the control group (Group G1) (Table 4). In contrast, NEUT values were significantly lower (*p* ≤ 0.05) in the G3 group than in G1 (i.e., control calves) (Table 4).

### 3.3. Gross Measurements of Rumen Tissue and Histomorphological Findings

A clear pattern of enhanced rumen development was observed across the experimental groups. Calves receiving 50 g of butyric salts (G3 group) exhibited significantly (*p* ≤ 0.001) longer rumen papillae and a markedly increased thickness of all rumen wall components, including the total rumen wall, muscular and serous layer, compared to both the G1 and G2 groups (Table 5).

Calves supplemented with 25 g of butyric salts (G2 group) also showed improvements, presenting significantly (*p* ≤ 0.05) longer rumen papillae and total rumen wall thickness (Table 5) than the control calves (G1 group). However, no significant differences were recorded between G2 and G1 groups in the thickness of the muscular or serous layers of the rumen wall (Table 5).

**Figure 3:**
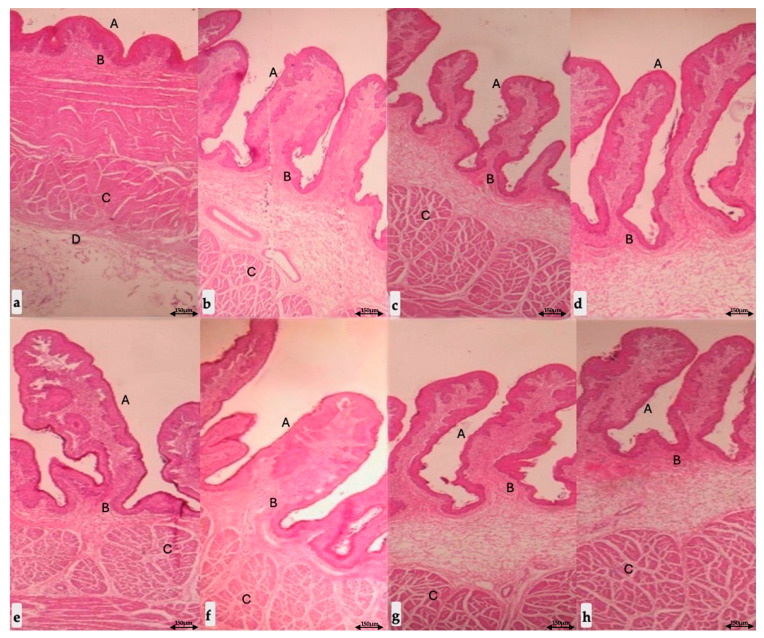
Histological findings of the rumen papillae of (i) the dorsal rumen sac between (**a**) calves (G1 group) fed with the standard diet and (**b**) calves (G3 group) fed the standard diet plus 50 g of butyric salts; (ii) the ventral rumen sac between (**c**) calves (G1 group) fed with the standard diet and (**d**) calves (G3 group) fed the standard diet plus 50 g of butyric salts; (iii) the caudodorsal rumen sac between (**e**) calves (G1 group) fed with the standard diet and (**f**) calves (G3 group) fed the standard diet plus 50 g of butyric salts; (iv) the caudoventral rumen sac between (**g**) calves (G1 group) fed with the standard diet and (**h**) calves (G3 group) fed the standard diet plus 50 g of butyric salts (stain: hematoxylin–eosin, magnification: 100×). A. Rumen papillae lined with a flat stratified epithelium. B. Cell layer underlying the areolar connective tissue lining epithelium. C. Internal circular muscular layer. D. Serous layer.

Representative histomorphological images illustrating these structural differences are presented in Figure 3.

### 3.4. Parasitological Findings

**Figure 4:**
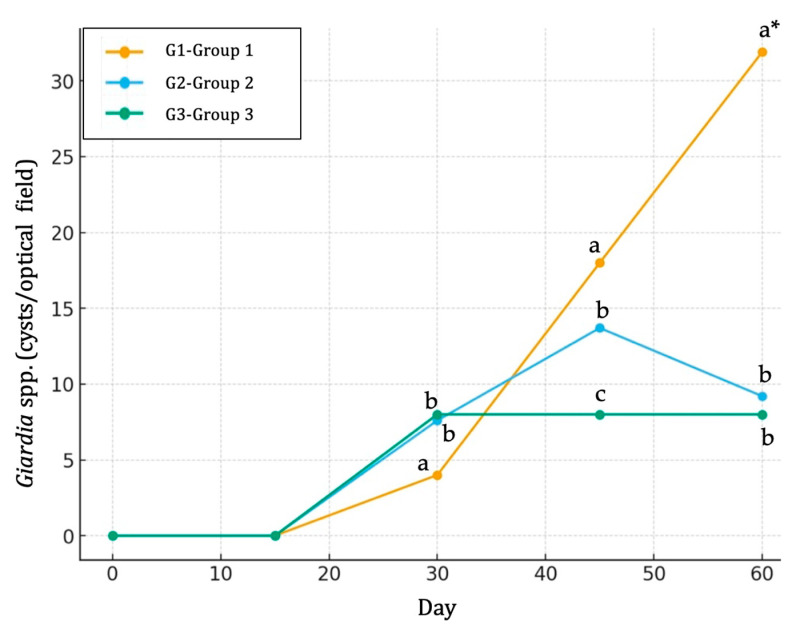
Line charts indicating the evolution and differences in *Giardia* spp. infection (cysts per field) among three groups (G1, G2 and G3) of calves across the experimental study. G1: group of calves (*n* = 10) fed with the standard diet; G2: group of calves (*n* = 10) fed with the standard diet plus 25 g of butyric salts; G3: group of calves (*n* = 10) fed with the standard diet plus 50 g of butyric salts. a, b and c: different letters indicate statistical differences at *p* ≤ 0.05 among groups per sampling occasion. * indicates statistical differences at *p* ≤ 0.001 among groups.

The dynamics of *Giardia* spp. infection varied notably among the experimental groups throughout the 60-day period (Table 6). In the control group (Group G1), *Giardia* spp. infection remained undetectable until Day 30, after which a sharp increase was observed, reaching a peak mean value of 31.9 by Day 60 (Figure 4). In contrast, calves from the G2 group exhibited a moderate rise, with cysts first detected on Day 30, peaking on Day 45, followed by a slight decline by Day 60 (Figure 4). Calves from the G3 group showed a stable pattern, characterized by low but consistent *Giardia* spp. cyst detection from Day 30 to Day 60 (Figure 4). Overall, a clear treatment effect was profound, with calves from the G1 group showing the highest (*p* ≤ 0.05) *Giardia* spp. burden compared to calves from both the G2 and G3 groups, while calves from the G3 group recorded the lowest (*p* ≤ 0.05) *Giardia* spp. excretion compared to the other two groups (Table 6).

**Figure 5:**
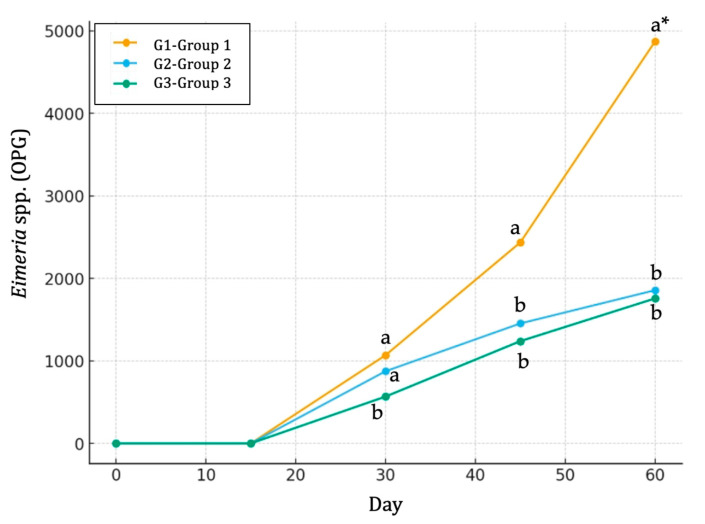
Line charts indicating the evolution and differences in *Eimeria* spp. infection (oocysts per g of feces, OPG) among three groups (G1, G2 and G3) of calves across the experimental study. G1: group of calves (*n* = 10) fed with the standard diet; G2: group of calves (*n* = 10) fed with the standard diet plus 25 g of butyric salts; G3: group of calves (*n* = 10) fed with the standard diet plus 50 g of butyric salts. a, b: different letters indicate statistical differences at *p* ≤ 0.05 among groups per sampling occasion. * indicates statistical differences at *p* ≤ 0.001 among groups.

No *Eimeria* spp. oocyst shedding was detected in any group on Day 0 and 15. By Day 30, infections became evident across all groups, with a prevalence ranging from 70% to 80%. Correspondingly, mean oocyst counts were significantly higher (*p* ≤ 0.05) in the control group (G1 group) compared to the G2 and G3 groups (Figure 5). By Day 45, the prevalence in the G1 group reached 100%, accompanied by a marked increase in oocyst output (2435.0 OPG). In contrast, the G2 and G3 groups maintained lower prevalence levels (70% and 90%, respectively) with correspondingly lower mean OPG values (1453.3 and 1238.6 OPG, respectively) (Figure 5). By Day 60, the prevalence reached 100% in both the G1 and G3 groups, while the G2 group remained at 80%. The highest oocyst burden was observed again in G1 (4870.0 OPG), which was more than twice the output recorded in both the G2 (1856.2 OPG) and G3 (1756.4 OPG) groups. Overall, the G1 group exhibited both the fastest rise in prevalence and the highest oocyst shedding throughout the experimental period (Figure 5). In contrast, the G2 and G3 groups showed comparatively lower and slower increases, suggesting differences in susceptibility or parasite dynamics between groups (Table 6).

## 4. Discussion

The present study aimed to evaluate the effects of butyric salt supplementations, applied as metabiotics, on rumen morphometric parameters and to examine the associations with feed intake, growth performance and health indicators, including a complete blood count, total plasma proteins and the susceptibility to two protozoan co-infections (*Giardia* spp. and *Eimeria* spp.) in pre-weaned dairy calves.

Metabiotics are microbially derived bioactive compounds that modulate host physiology without requiring live microorganisms [11]. Among them, butyric salts are of increasing interest because butyrate, their primary active compound, serves both as a metabolic substrate and as a key signaling molecule for rumen epithelial cells. Butyrate promotes epithelial proliferation, differentiation and vascularization, thereby enhancing papillary growth, increasing rumen absorptive surface area and improving the capacity for VFA uptake [12].

In the present study, pre-weaned dairy calves supplemented with butyric salts, either with 25 g or 50 g, produced marked, dose-dependent increases in rumen papillae length and in total rumen wall thickness (including muscular and serosal layers). These findings are consistent with previous reports showing that butyric salts stimulate rumen papillae development, enhance nutrient absorption, modulate mucosal immunity and support the transition from liquid to solid feed, thereby improving growth performance in young ruminants [13,14].

Rumen morphometry, however, is influenced by several additional factors beyond butyrate exposure. Diet composition is among the most influential determinants of papillary growth. More precisely, starch-rich, highly fermentable concentrates stimulate amylolytic bacterial populations and VFA production, particularly butyric acid, which in turn promotes papillae growth [20,21]. Intensive concentrate feeding has been consistently associated with greater papillary development in beef cattle. Reddy et al. [22] reported significant increases in papilla length, width, mucosal thickness and overall rumen wall thickness across multiple anatomical regions in Korean beef cattle fed high-concentrate diets, with magnitudes of change comparable to those observed in the present study [22].

In contrast to concentrate-based diets, forage-rich rations are generally associated with a reduced papillary length and slower epithelial development [23]. Nevertheless, strategic forage inclusion during the pre-weaning period can be beneficial. For example, supplementation with oat hay has been shown to enhance rumen function by increasing the total dry matter intake and rumination rate, stabilizing rumen pH, reducing abnormal oral behaviors and supporting healthy mucosal maturation [24]. Similarly, incorporating alfalfa hay into starter feeds has been associated with improved calf growth performance despite a higher total feed intake [25]. Collectively, these findings indicate that both the concentrate composition and the timing and type of forage inclusion shape the trajectory of rumen morphological maturation, consistent with the patterns observed in our study. Notably, Novak et al. [26] found no significant differences in papillae length or width in Jersey steers fed diets differing in forage-to-concentrate ratios, highlighting that species, breed and management contexts can modulate the dietary effects on rumen morphometric outcomes [26].

The physical form of concentrates also affects rumen papillae size by modifying the balance between cellulolytic and amylolytic bacterial populations [27]. Castro-Flores and Elizondo-Salazar [28] compared powder, pelleted and extruded starter concentrates in 60-day-old pre-weaned calves and reported that powder-form concentrates produced the largest rumen papillae and the thickest rumen wall. These findings highlight that not only nutrient composition but also physical characteristics of the diet modulate rumen epithelial growth during early life.

Animals’ age and the timing of solid feed introduction are additional key determinants of rumen morphometric development. The rumen papillae length increases progressively with age as well as with chemical stimulation from VFA, and the early provision of fermentable solid feed induces papillary growth proportional to the rate of intake increase [5,29,30]. Thus, the differences in sampling age can explain discrepant measurements between studies. For example, Zitnan et al. [31] reported smaller papillary dimensions at 41 days of age compared with those recorded in the present study at 60 days, a discrepancy likely attributable to the earlier stage of rumen development at their sampling occasion. Likewise, early starter diets that accelerate the intake of fermentable substrates contribute to a higher average daily gain and improved rumen development in young calves [6,32].

Endocrine factors, such as colostrum-derived growth factors, can also influence rumen development [33]. In our experimental study, all groups received the same base diet, including concentrates and roughages along with identical colostrum and milk management. The principal dietary difference among the groups was the graded supplementation of butyric salts. Therefore, the observed effects on rumen morphometry are consistent with enhanced rumen fermentation and the consequent morphological development driven specifically by butyric salt intake [34,35,36].

In our study, calves receiving the highest level of butyric supplementation (50 g) exhibited a pronounced rumen development compared to those receiving the lower dose (25 g). Particularly, the mean rumen wall thickness reached 7.08 mm, and the mean papillary height was 3.20 mm. These dimensions exceeded those reported by Castro-Flores and Elizondo-Salazar [28], who measured papilla lengths of 1.93 mm and wall thicknesses of 2.62 mm in calves fed powder-form concentrates at 60 days of age. The latter authors attributed their findings to increased endogenous butyric acid production from highly digestible carbohydrates in the concentrate mix (around 51%).

It is important to recognize that excessive fermentation or inappropriate feeding strategies can adversely affect the stratified squamous epithelium of the rumen. High fermentative activity, low rumen pH, elevated osmolarity, microbial toxins and associated immune mediators may lead to epithelial lesions and, in severe cases, systemic inflammation. While butyrate is beneficial during early rumen development, excessive exposure has been implicated in hyperkeratosis, parakeratosis and epithelial damage in the fully developed rumen of adult cattle [37]. Therefore, a careful optimization of the type, form and quantity of fermentable substrates, alongside balanced butyric salt supplementation, is a key essential for promoting healthy rumen maturation without inducing pathological changes.

The dose-dependent improvements in papillae height and rumen wall thickness observed here align with the established trophic actions of butyrate on the ruminant forestomach. Butyrate is the preferred oxidative substrate for rumen epithelial cells, taken up largely via monocarboxylate transporters and rapidly oxidized, which provides ATP for the growth and differentiation of the stratified squamous epithelium [12]. The experimental infusion or dietary delivery of butyrate upregulates epithelial cell cycle drivers, including transient increases in cyclin D1, and stimulates angiogenesis within the papillary core, changes that expand the absorptive surface area and enhance VFA clearance from the rumen [13,14]. These cellular effects are consistent with our morphometric data, particularly in the G3 group, and provide a mechanistic basis for a greater VFA uptake per unit of time, faster functional adaptation to solid feed and the superior dry matter intake, average daily gain and feed conversion ratio that we recorded. Beyond its trophic role, butyrate supports epithelial integrity. Work in ruminants and non-ruminant models has shown that butyrate strengthens tight junction assembly through AMPK signaling and contributes to a more resilient mucosal barrier that limits the penetration of luminal antigens and toxins [37,38]. Barrier reinforcement reduces local inflammation and the energetic cost of immune activation, which can translate into improved nutrient partitioning toward growth. This mechanism, together with the larger absorptive surface, offers a coherent explanation for the improved efficiency in G2 and especially G3 animals.

*Eimeria* spp. and *Giardia* spp. are prevalent enteric protozoa in pre-weaned dairy calves, posing significant health challenges. *Eimeria* spp. can lead to reduced growth rates, poor feed conversion and even mortality in severe cases [39]. Similarly, *Giardia* spp. infections have been associated with a decreased average daily gain and increased susceptibility to diarrhea [40]. These infections can compromise gut health and immune function, leading to the increased use of antimicrobial and antiparasitic drugs and potential long-term performance issues. Therefore, these two protozoal infections represent a critical concern for calf health and futural productivity under intensive management systems.

In our study, the parasitological results were, also, notable. Calves who received butyric salts had a lower *Giardia* spp. and *Eimeria* spp. (oo)cyst excretion than the controls throughout the experimental period. Several non-mutually exclusive mechanisms may account for these findings. An improved rumen maturation, enhanced mucosal barrier function and local immune modulation induced by butyrate could reduce protozoan parasite establishment and fecal shedding [12,41]. In addition, more rapid gut maturation driven by a higher solid feed intake may shorten the window of vulnerability to enteric pathogens [42]. Shifts in gut microbiota composition and fermentation, although not measured in this study, could also contribute to an environment less favorable for parasite proliferation [38,43]. The reduced (oo)cyst counts observed in calves receiving butyric salts may also reflect an improved allocation of metabolic resources toward immune system development. The increased feed intake and nutrient digestibility recorded in supplemented animals suggest a greater availability of energy and amino acids to support immune functions, including local intestinal immunity against protozoan parasites. Consequently, the immune system may have been better equipped to control infections and limit parasite replication. These data suggest an ancillary benefit of butyric salt supplementation in reducing enteric protozoan parasitism. However, cause–effect relationships should be explored with targeted immunological and microbiome analyses. No clinical signs of these two protozoan infections were observed in any calves during the experimental period. As all animals were slaughtered at weaning, clinical progression beyond this age could not be assessed. The enumeration of *Eimeria* and *Giardia* (oo)cysts was therefore used as an indicator of intestinal parasitic burden rather than clinical disease expression. It is also important to note that the butyric acid source used in this study was sodium butyrate, which is rapidly absorbed in the upper gastrointestinal tract. While encapsulated or protected forms can reach the distal gut and exert localized effects, the systemic improvement in intestinal health observed here indicates that even non-encapsulated butyrate can indirectly enhance gut immune competence and epithelial defense mechanisms.

Hematological changes in butyric salt-fed calves further support a beneficial systemic effect. At Day 60, calves in the highest supplementation group (G3) had higher PCV and HGB concentrations and increased MCHC. Leukocyte differentials shifted toward higher monocyte percentages and lower neutrophil percentages at weaning. The observed hematological trends, particularly the higher HGB concentration and lower NEUT proportion in calves receiving butyric salts, may reflect an improved systemic oxygen transport and a reduction in inflammatory or stress-related responses. Butyrate has been shown to influence cellular metabolism and gene expression in multiple tissues through histone deacetylase inhibition and the activation of AMP-activated protein kinase pathways [12,44]. These mechanisms can enhance epithelial and systemic energy efficiency and may also support erythropoiesis, contributing to the higher HGB concentrations observed in supplemented calves. Furthermore, butyrate exerts anti-inflammatory and immunomodulatory effects by reinforcing epithelial barrier integrity, modulating cytokine expression and promoting regulatory immune cell differentiation [45,46,47]. This could explain the reduced NEUT counts and higher MONO levels detected at weaning, indicating a more balanced immune response and lower systemic stress.

Based on these results, several priorities for future research emerge. First, experiments with larger and more diverse calf populations, under different feeding systems and across both sexes, are required to confirm external validity and to determine the economic return on supplementation. Second, complementary measurements of rumen fermentation (VFA concentrations and profiles), mucosal transcriptomics (cell proliferation markers, transporters, inflammatory mediators) and microbiome composition would elucidate mechanisms linking butyrate to epithelial growth, nutrient absorption and parasite resistance. Although quantifying rumen butyrate could have provided additional mechanistic data, such measurements were beyond the scope of this study, which aimed primarily to assess growth performance, rumen development and health outcomes. Moreover, given the rapid metabolism and absorption of exogenous butyrate along the gastrointestinal tract, rumen concentrations alone may not accurately reflect the physiological exposure of target tissues. Third, dose–response trials comparing different butyrate salts (e.g., sodium versus calcium butyrate), the timing of initiation and duration of supplementation and potential interactions with milk-feeding programs will help optimize practical recommendations. Finally, longitudinal studies following animals through weaning and into first lactation/performance are needed to determine whether early-life metabiotic interventions confer durable productivity benefits.

In practical terms, our findings indicate that butyric salts administered from birth to weaning can accelerate rumen development, improve intake and growth rates and reduce enteric protozoan burdens under on-field conditions. If these findings are confirmed under larger-scale and variable commercial conditions, this approach could meaningfully improve calf welfare and productivity while reducing the reliance on therapeutic antimicrobial and antiprotozoal drugs, an important target for sustainable livestock production. Caution is warranted regarding routine on-farm use, particularly with respect to dose optimization and monitoring for potential adverse effects from excessive fermentation. Future multi-site, long-term studies will be essential to validate these results and establish practical guidelines for safe and effective implementation.

## 5. Conclusions

Supplementation with sodium butyrate in pre-weaned dairy calves produced clear, dose-dependent improvements in rumen morphometry, voluntary dry matter intake and growth performance. These physiological benefits were accompanied by the reduced shedding of *Giardia* spp. and *Eimeria* spp. (oo)cysts, along with favorable shifts in hematological parameters indicative of an enhanced immune competence. Our findings support the use of butyric salts as a metabiotic factor to promote early rumen maturation and overall health in dairy calves. Moreover, this approach may also help reduce the reliance on therapeutic antimicrobial and antiparasitic drugs, aligning with the objectives of sustainable and responsible livestock production.

## Figures and Tables

**Table 1:** Daily feeding of whole milk or milk replacer by week.

Week	WM/MR (L)
1st	4.5–6.0
2nd	7.0
3rd	7.0
4th	7.0
5th	7.0
6th	7.0
7th	5.0
8th	2.0
9th	0.0

WM: whole milk, MR: milk replacer, L: liters.

**Table 2:** Effects of butyric salts on body weight, body weight gain, dry matter intake and feed conversion ratio of calves across the experimental study.

	Treatment ^1^			SEM
	G1	G2	G3	
Initial BW (day of birth, kg)	47.1 ^a^	47.4 ^a^	46.0 ^a^	1.10
Final BW (60 day of age, kg)	80.1 ^a^	87.3 ^b^	93.9 ^c^	1.73
BW gain (g/day)	550 ^a^	665 ^b^	798 ^c,^*	71.66
Concentrate mixture (expressed as DM intake, kg)	15.6 ^a^	19.2 ^b^	24.7 ^c^	2.65
Milk replacer (expressed as DM intake, kg)	35.9 ^a^	36.4 ^a^	38.5 ^a^	0.79
Alfalfa hay (expressed as DM intake, kg)	3.6 ^a^	5.1 ^a^	6.2 ^b^	0.75
Sum of total DM intake ^2^ (kg)	55.1 ^a^	60.7 ^a^	69.4 ^b^	4.16
FCR (kg DM intake/kg BW gain)	1.67 ^a^	1.52^b^	1.45 ^c,^*	0.07

^1^ G1: group of calves (*n* = 10) fed with the standard diet; G2: group of calves (*n* = 10) fed with the standard diet plus 25 g of butyric salts; G3: group of calves (*n* = 10) fed with the standard diet plus 50 g of butyric salts. ^2^ Calves were fed with concentrate mixture and milk replacer during the whole experimental period (8–60 days of age) and with alfalfa hay from days 31 to 60 of age. BW: body weight, DM: dry matter, FCR: feed conversion ratio. ^a, b^ and ^c^: different superscripts indicate statistical differences at *p* ≤ 0.05 among groups. * indicates statistical differences at *p* ≤ 0.001 among groups.

**Table 3:** Effects of butyric salt supplementation on complete blood count and total proteins among three groups (G1, G2 and G3) of calves until roughage provision (30 days of age).

Hematological Parameters	Reference Ranges ^1^	Treatment ^2^			SEM
		G1	G2	G3	
Hematocrit (PCV, %)	24–46	26.0 ^a^	27.1 ^a^	30.7 ^b^	1.27
Hemoglobin (HGB, n × 10 g/L)	8–15	10.1 ^a^	9.4 ^a^	10.0 ^a^	0.46
Red blood cells (RBC, n × 10^12^/L)	5–10	7.4 ^a^	6.8 ^a^	7.8 ^a^	0.30
Mean corpuscular volume (MCV, fL = 10^−15^ L)	40–60	37.9 ^a^	37.8 ^a^	39.6 ^a^	0.56
Mean corpuscular hemoglobin (MCH, pg)	11–17	12.0 ^a^	13.0 ^a^	13.4 ^a^	0.39
Mean corpuscular hemoglobin concentration (MCHC, n × 10 g/L)	30–36	32.1 ^a^	33.4 ^a^	33.7 ^a^	0.56
Red cell distribution width (RDW, %)	15.5–19.7	21.5 ^a^	22.0 ^a^	20.2 ^a^	0.57
Platelets (n × 10^9^/L)	100–800	597 ^a^	628 ^a^	432 ^a^	47.9
Mean platelet volume (MPV, fL = 10^−15^ L)	3.5–6.5	6.2 ^a^	6.4 ^a^	7.3 ^a^	0.20
White blood cells (WBC, n × 10^9^/L)	4–12	9.1 ^a^	10.2 ^a^	10.2 ^a^	0.58
Neutrophils (%)	15–33	47.3 ^a^	48.9 ^a^	55.9 ^b^	1.96
Neutrophils (n × 10^9^/L)	0.6–4.0	4.3 ^a^	4.1 ^a^	5.8 ^a^	0.43
Lymphocytes (%)	45–75	39.5 ^a^	34.9 ^a^	34.4 ^a^	1.77
Lymphocytes (n × 10^9^/L)	2.5–7.5	3.7 ^a^	3.4 ^a^	3.4 ^a^	0.26
Monocytes (%)	0–8	3.2 ^a^	3.5 ^a^	6.7 ^b^	0.56
Monocytes (n × 10^9^/L)	0–0.9	0.3 ^a^	0.3 ^a^	0.7 ^b^	0.06
Eosinophils (%)	0–20	5.6 ^b^	1.9 ^a^	2.0 ^a^	0.61
Eosinophils (n × 10^9^/L)	0–2.4	0.4 ^b^	0.2 ^a^	0.2 ^a^	0.04
Basophils (%)	0–2	0.3 ^a^	0.5 ^a^	1.0 ^b^	0.06
Basophils (n × 10^9^/L)	0–0.2	0.1 ^a^	0.1 ^a^	0.1 ^a^	0.01
Total proteins (g/dL)	5.7–8.1	6.6 ^a^	6.7 ^a^	7.8 ^b^	0.19

^1^ Adapted from Merck Veterinary Manual [18]. Reference ranges for RDW and total proteins were adapted from Constable et al. [19]. ^2^ G1: group of calves (*n* = 10) fed with the standard diet; G2: group of calves (*n* = 10) fed with the standard diet plus 25 g of butyric salts; G3: group of calves (*n* = 10) fed with the standard diet plus 50 g of butyric salts. SD, SEM: standard error of the mean. ^a, b^: different superscripts in each row indicate statistical differences at *p* ≤ 0.05 among groups per hematological parameter.

**Table 4:** Effects of butyric salt supplementation on complete blood count and total proteins among three groups (G1, G2 and G3) of calves at weaning (60 days of age).

Hematological Parameters	Reference Ranges ^1^	Treatment ^2^			SEM
		G1	G2	G3	
Hematocrit (PCV, %)	24–46	28.8 ^a^	35.6 ^b^	32.6 ^b^	0.96
Hemoglobin (HGB, n × 10 g/L)	8–15	10.6 ^a^	11.7 ^b^	11.9 ^b^	0.20
Red blood cells (RBC, n × 10^12^/L)	5–10	11.6 ^a^	11.9 ^a^	11.1 ^a^	0.18
Mean corpuscular volume (MCV, fL = 10^−15^ L)	40–60	29.3 ^a^	30.8 ^a^	30.7 ^a^	0.47
Mean corpuscular hemoglobin (MCH, pg)	11–17	11.1 ^a^	10.6 ^a^	11.2 ^a^	0.30
Mean corpuscular hemoglobin concentration (MCHC, n × 10 g/L)	30–36	31.7 ^a^	33.9 ^a,b^	35.7 ^b^	0.50
Red cell distribution width (RDW, %)	15.5–19.7	20.5 ^a^	22.0 ^a^	21.8 ^a^	0.38
Platelets (n × 10^9^/L)	100–800	508 ^a^	582 ^a^	565 ^a^	22.5
Mean platelet volume (MPV, fL = 10^−15^ L)	3.5–6.5	6.3 ^a^	6.3 ^a^	6.6 ^a^	0.21
White blood cells (WBC, n × 10^9^/L)	4–12	11.1 ^a^	11.4 ^a^	10.5 ^a^	0.49
Neutrophils (%)	15–33	39.0 ^a^	34.3 ^a^	27.7 ^b^	2.32
Neutrophils (n × 10^9^/L)	0.6–4.0	4.3 ^a^	4.3 ^a^	3.0 ^a^	0.42
Lymphocytes (%)	45–75	52.5 ^a^	56.5 ^a^	60.3 ^a^	2.02
Lymphocytes (n × 10^9^/L)	2.5–7.5	5.9 ^a^	6.1 ^a^	6.2 ^a^	0.14
Monocytes (%)	0–8	5.4 ^a^	5.9 ^a^	8.3 ^b^	0.60
Monocytes (n × 10^9^/L)	0–0.9	0.6 ^a^	0.7 ^a^	0.8 ^a^	0.05
Eosinophils (%)	0–20	1.6 ^a^	1.7 ^a^	1.9 ^a^	0.16
Eosinophils (n × 10^9^/L)	0–2.4	0.2 ^a^	0.2a	0.2 ^a^	0.02
Basophils (%)	0–2	1.4 ^a^	1.6 ^a^	1.8 ^a^	0.15
Basophils (n × 10^9^/L)	0–0.2	0.2 ^a^	0.2 ^a^	0.2 ^a^	0.02
Total proteins (g/dL)	5.7–8.1	6.7 ^a^	6.8 ^a^	6.7 ^a^	0.18

^1^ Adapted from Merck Veterinary Manual [18]. Reference ranges for RDW and total proteins were adapted from Constable et al. [19]. ^2^ G1: group of calves (*n* = 10) fed with the standard diet; G2: group of calves (*n* = 10) fed with the standard diet plus 25 g of butyric salts; G3: group of calves (*n* = 10) fed with the standard diet plus 50 g of butyric salts. SD: standard deviation, SEM: standard error of the mean. ^a, b^: different superscripts in each row indicate statistical differences at *p* ≤ 0.05 among groups per hematological parameter.

**Table 5:** Measurements (millimeters) of rumen papillae length and thickness of the wall, muscular and serous layer in the four anatomical regions of the rumen tissue in *Holstein* calves at weaning (Day 61) among three groups (G1, G2 and G3).

Rumen		Group	Anatomical Region				Mean (mm)	SD
Parameter	Structure		Dorsal Sac (mm)	Ventral Sac (mm)	Caudodorsal Sac (mm)	Caudoventral Sac (mm)		
Length	Rumen papillae	G1 (*n* = 10)	1.00	1.55	1.44	1.44	1.36 ^a^	0.244
		G2 (*n* = 10)	1.55	1.95	1.94	2.21	1.91 ^b^	0.272
		G3 (*n* = 10)	2.60	3.57	3.12	3.50	3.20 ^c,^*	0.444
Thickness	Wall	G1 (*n* = 10)	3.54	3.46	3.51	4.00	3.62 ^a^	0.251
		G2 (*n* = 10)	4.32	5.34	5.34	5.23	5.06 ^b^	0.494
		G3 (*n* = 10)	5.87	8.06	6.87	7.51	7.08 ^c,^*	0.941
	Muscular	G1 (*n* = 10)	2.00	1.43	1.53	2.00	1.74 ^a^	0.303
		G2 (*n* = 10)	2.14	1.98	1.66	2.57	2.09 ^a^	0.378
		G3 (*n* = 10)	2.50	3.02	2.89	3.49	2.97 ^b,^*	0.408
	Serous	G1 (*n* = 10)	0.50	0.50	0.53	0.50	0.51 ^a^	0.015
		G2 (*n* = 10)	0.66	0.60	0.59	0.60	0.61 ^a^	0.032
		G3 (*n* = 10)	0.99	1.00	1.02	1.00	1.00 ^b,^*	0.013

G1: group of calves (*n* = 10) fed with the standard diet; G2: group of calves (*n* = 10) fed with the standard diet plus 25 g of butyric salts, G3; group of calves (*n* = 10) fed with the standard diet plus 50 g of butyric salts. SD: standard deviation, mm: millimeters. ^a, b^ and ^c^: different superscripts indicate statistical differences at *p* ≤ 0.05 among groups per parameter and structure of the rumen. * indicates statistical differences at *p* ≤ 0.001 among groups per parameter and structure of the rumen.

**Table 6:** Prevalence of *Giardia* spp. and *Eimeria* spp. infection and mean (±SD) number of (oo)cyst enumeration, respectively, among groups per sampling occasion.

Group	Day	*Giardia* spp.								*Eimeria* spp.			
		Prevalence (%)		Score				Mean	SD	Prevalence (%)		Mean	SD
				1	2	3	4	(cysts/Optical Field)				(OPG)	
G1	0	0.0	0/10	0	0	0	0	0.0 ^a^	0.00	0.0	0/10	0.0 ^a^	0.00
	15	0.0	0/10	0	0	0	0	0.0 ^a^	0.00	0.0	0/10	0.0 ^a^	0.00
	30	40.0	4/10	4	0	0	0	4.0 ^a^	0.50	80.0	8/10	1068.8 ^a^	168.89
	45	60.0	6/10	4	2	0	0	18.0 ^a^	15.37	100.0	10/10	2435.0 ^a^	515.35
	60	80.0	8/10	3	4	1	0	31.9 ^a,^*	23.79	100.0	10/10	4870.0 ^a,^*	1072.43
G2	0	0.0	0/10	0	0	0	0	0.0 ^a^	0.00	0.0	0/10	0.0 ^a^	0.00
	15	0.0	0/10	0	0	0	0	0.0 ^a^	0.00	0.0	0/10	0.0 ^a^	0.00
	30	30.0	3/10	3	0	0	0	7.6 ^b^	0.57	70.0	7/10	874.4 ^a^	145.34
	45	60.0	6/10	5	1	0	0	13.7 ^b^	11.23	70.0	7/10	1453.3 ^b^	187.34
	60	60.0	6/10	5	1	0	0	9.2 ^b^	2.93	80.0	8/10	1856.2 ^b^	123.35
G3	0	0.0	0/10	0	0	0	0	0.0 ^a^	0.00	0.0	0/10	0.0 ^a^	0.00
	15	0.0	0/10	0	0	0	0	0.0 ^a^	0.00	0.0	0/10	0.0 ^a^	0.00
	30	40.0	4/10	4	0	0	0	8.0 ^b^	0.82	70.0	7/10	567.3 ^b^	123.23
	45	50.0	5/10	5	0	0	0	8.0 ^c^	0.71	90.0	9/10	1238.6 ^b^	110.45
	60	10.0	1/10	1	0	0	0	8.0 ^b^	0.00	100.0	10/10	1756.4 ^b^	86.67

G1: group of calves (*n* = 10) fed with the standard diet; G2: group of calves (*n* = 10) fed with the standard diet plus 25 g of butyric salts; G3: group of calves (*n* = 10) fed with the standard diet plus 50 g of butyric salts. [score: +1] 1–9 cysts, [score: +2] 10–50 cysts, [score: +3] 51–100 cysts and [score: +4] >100 cysts. OPG: oocysts per g of feces, SD: standard deviation. ^a, b^ and ^c^: different superscripts indicate statistical differences at *p* ≤ 0.05 among groups per sampling occasion. * indicates statistical differences at *p* ≤ 0.001 among groups.

## Data Availability

The datasets used and/or analyzed during the current study are available from the corresponding author upon reasonable request.
